# Rare gastrointestinal stromal tumor in an ileal conduit detected by recurrent massive bleeding from the stoma

**DOI:** 10.1002/iju5.12190

**Published:** 2020-08-04

**Authors:** Michio Noda, Yukimasa Matsuzawa, Hiroaki Nishimatsu, Shinichiro Murayama, Hirohisa Kishi, Masaki Nakamura, Haruki Kume

**Affiliations:** ^1^ Department of Urology The Fraternity Memorial Hospital Tokyo Japan; ^2^ Department of Urology Graduate School of Medicine The University of Tokyo Tokyo Japan; ^3^ Department of Pathology The Fraternity Memorial Hospital Tokyo Japan

**Keywords:** gastrointestinal stromal tumor, ileal conduit, radical cystectomy

## Abstract

**Introduction:**

The development of secondary tumors is a well‐known late adverse event after urinary diversion. However, the frequency of secondary tumors after an ileal conduit is the lowest compared to other methods used for urinary diversion. We observed a rare case of a gastrointestinal stromal tumor in an ileal conduit detected by recurrent massive bleeding from the stoma.

**Case presentation:**

An 87‐year‐old female was hospitalized at our hospital due to recurrent bleeding from a stoma 22 years after radical cystectomy. Contrast‐enhanced computed tomography revealed a 5‐cm mass in her ileal conduit. She underwent a complete resection of the tumor, a histological examination of which revealed it to be a gastrointestinal stromal tumor. The condition of the patient has been good showing no recurrence or metastases 4 years after surgery.

**Conclusion:**

We report a rare secondary tumor, a gastrointestinal stromal tumor, arising from an ileal conduit.

**Abbreviations & Acronyms**
CT: computed tomography
GIST: gastrointestinal stromal tumor
Hb: hemoglobin
HE: hematoxylin and eosin
HPF: high‐powered field
SMA: smooth muscle actin

**Keynote message**

We observed a case of a GIST in an ileal conduit detected after recurrent massive bleeding from a stoma. Although the development of secondary tumors is well known as late adverse event after urinary diversion, a GIST has been rarely reported as a secondary tumor.

## Introduction

Radical cystectomy is the standard treatment for invasive urothelial cell carcinoma of the bladder and an ileal loop conduit has long been the most common method for urinary diversion after cystectomy.^1^

The development of secondary tumors is well known as a late adverse event after urinary diversion. However, the prevalence of tumor development after an ileal conduit was reported to be minimal compared to the other urinary diversions in a multicenter analysis.^2^ In addition, the histopathology of most secondary tumors are adenomas and adenocarcinomas.^2^, ^3^

We present an 87‐year‐old female who was admitted to our hospital with anemia due to recurrent bleeding from a protruding tumor detected in her stoma. The pathology of this tumor was finally revealed to be a GIST. This is the first reported case of a GIST in an ileal conduit.

## Case presentation

An 87‐year‐old female underwent a radical cystectomy with an ileal conduit for invasive bladder cancer. Pathological examination demonstrated G3 urothelial carcinoma with a component of squamous cell carcinoma (<5%), pT3b. she underwent two cycles of adjuvant chemotherapy with methotrexate, vinblastine, doxorubicin, and cisplatin and was in good condition without metastatic disease.

**Fig. 1:**
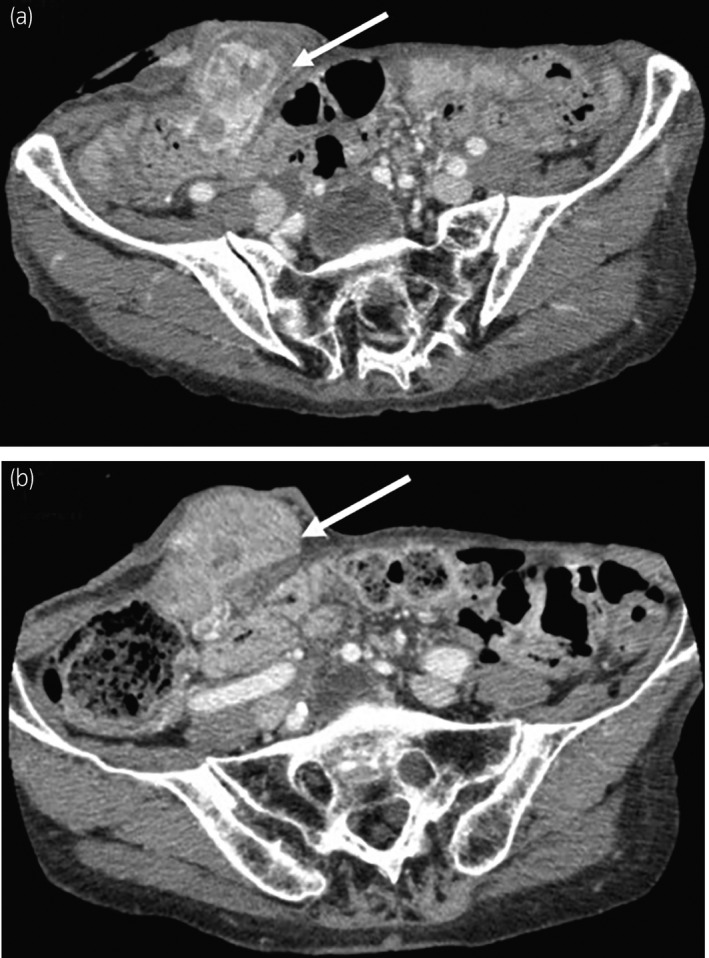
A white arrow points to: (a) a 35 × 30 × 25 mm mass detected by enhanced CT on the first admission with bleeding; and (b) a 50 × 45 × 40 mm mass detected by enhanced CT 3 years later.

Nineteen years after the cystectomy, she was admitted to our hospital with anemia and Hb of 8 g/dL. Bleeding from the stoma continued and contrast‐enhanced CT showed a tumor measuring 35 × 30 × 25 mm on the right‐side wall of the conduit (Fig. 1a). Biopsy examination of the tumor revealed erosive mucosa and granulation tissue without malignancy. The bleeding from the stoma stopped with conservative therapy and she refused invasive treatment at the time; she was therefore discharged after 13 days’ hospitalization. After hospitalization, she underwent outpatient checkups every 3 months, with no recurrence of bleeding thereafter.

Three years later, acute bleeding from the stoma recurred and she was hospitalized again with severe anemia (Hb of 5.9 g/dL). Contrast‐enhanced CT revealed an enlarged tumor measuring 50 × 45 × 40 mm in the ileal conduit (Fig. 1b).

The bleeding did not stop this time with conservative therapy and the size of this tumor had been gradually increasing over 3 years, so she underwent a complete resection of the tumor 16 days after admission.

**Fig. 2:**
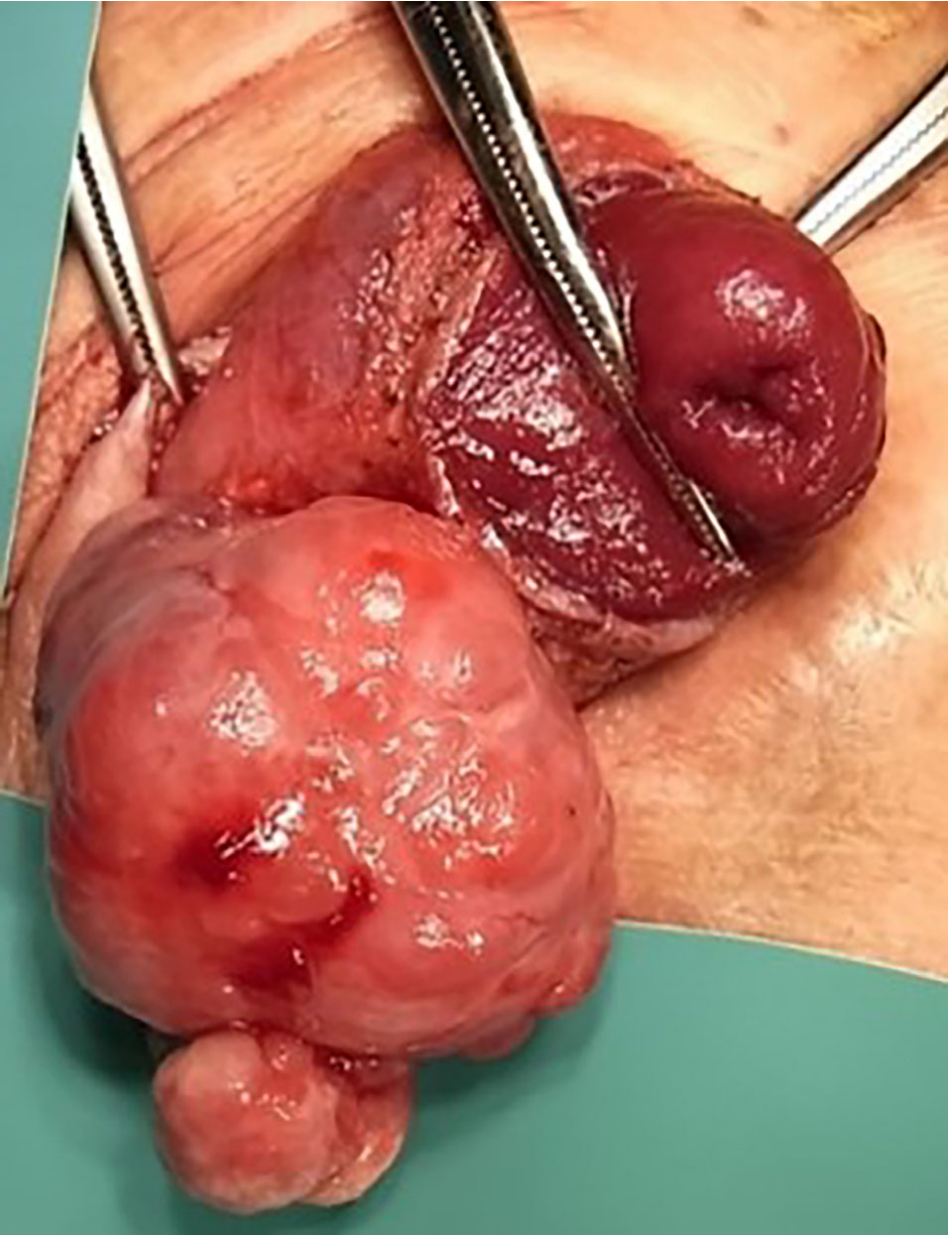
A solid and multinodular 50 × 45 × 40 mm‐sized tumor was resected.

**Fig. 3:**
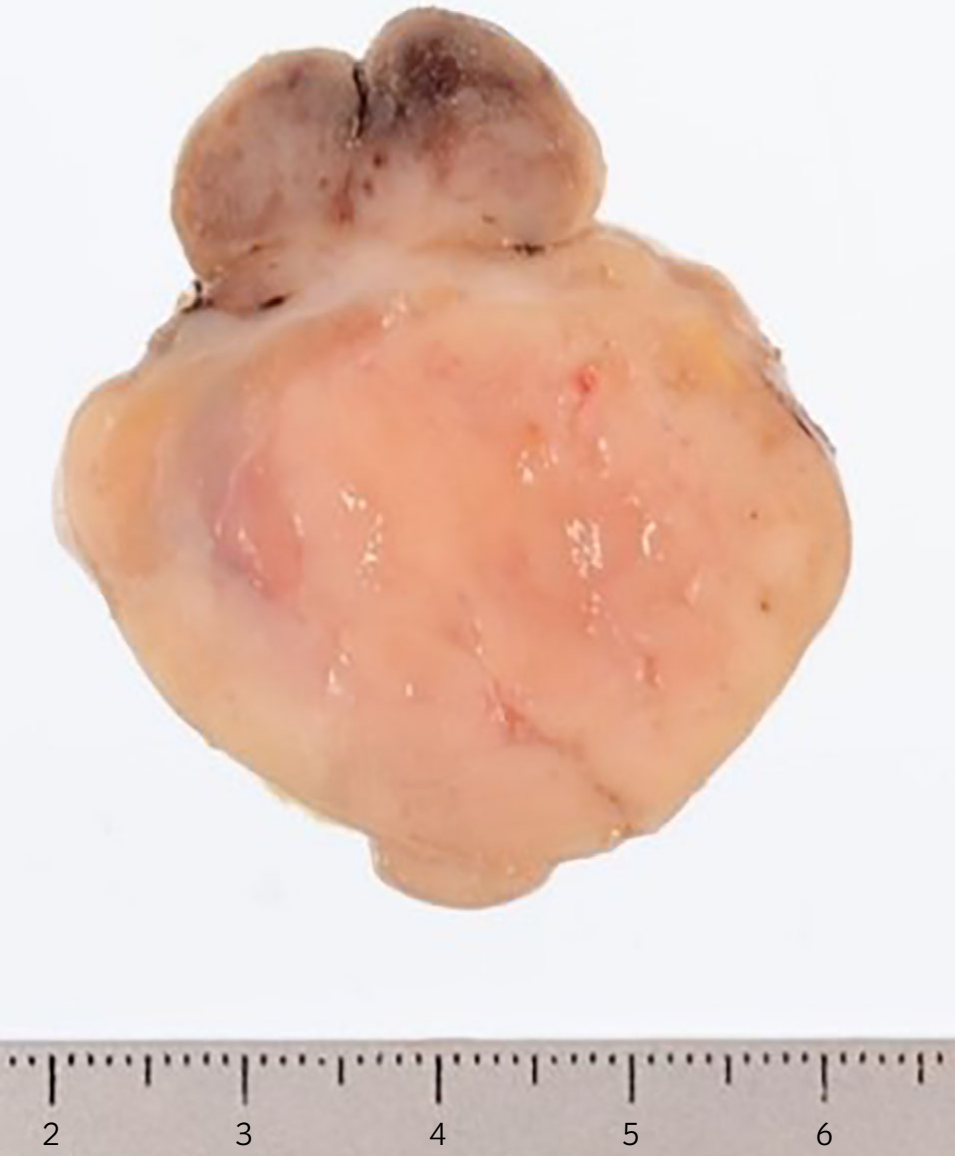
The cut surface of the tumor was light gray in color.

**Fig. 4:**
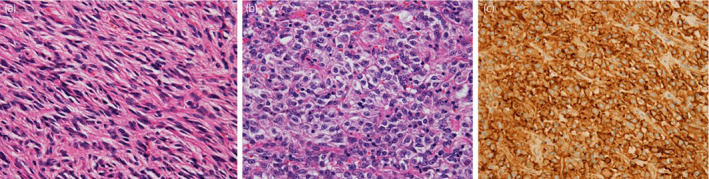
Microscopic appearance. (a) The tumor was mostly composed of irregularly arranged spindle cells (HE). (b) Rounded cells were partially detected in the tumor (HE). (c) CD117 (*KIT*) immunoreactivity in tumor cells.

The tumor was recognized from the surface of her abdominal wall under general anesthesia. Five centimeters incision was made just beside the stoma on the opposite side of mesentery and the tumor was dragged out of the stoma. The mass was not adhered to its surrounding tissue and we performed en bloc resection of the tumor with its stalk from the wall of conduit. It was a solid and multinodular 50*45*40‐mm‐sized tumor, mostly covered with mucosa of the small intestine (Fig. 2). The cut surface of this tumor was light gray in color (Fig. 3). Surgical margin was histologically negative, therefore her conduit was preserved. Histopathological examination showed the tumor consisted of irregularly arranged spindle cells as well as rounded cells (Fig. 4). The mitotic count was below 5 per 50 HPF.

Immunohistochemical examination revealed reactivity of CD117 (*KIT*) and SMA antibodies. However, CD34 and S100 were not stained. The tumor was histopathologically diagnosed as a GIST (low‐risk type).

The patient was discharged in a good condition 11 days after surgery without any adverse post‐operative events. She has had no recurrence or metastasis 4 years since the tumor was resected.

## Discussion

Many types of urinary diversions exist, including ureterocutaneostomy, ileal conduit, and orthotopic neobladder reconstruction. The development of secondary tumors after urinary diversion is a well‐recognized late adverse event. It has been reported that adenocarcinomas are the most common pathological type, followed by adenomas.^2^, ^3^ Although other types of secondary tumors, such as urothelial carcinomas, carcinoid, and squamous cell carcinomas, have been reported, the development of a GIST has been reported in only one case, in which GIST developed in a neobladder 4 years after neobladder reconstruction.^4^ We are the first to report the development of a GIST in an ileal conduit.

The pathogenesis of secondary tumors after urinary diversion is not well understood. Inflammatory changes and N‐nitrosamine formation induced by chronic exposure of the bowel to urine are reported to be possible mechanisms.^5^, ^6^ N‐nitrosamine has been suggested to initiate the multistage process of neoplastic transformation by causing irreversible changes in DNA structure.^7^ Other possible mechanisms, such as surgical and mechanical trauma, an excess concentration of electrolytes, and exposure to free radicals, are recognized.^8^

An article which has summarized the prevalence of secondary tumor growth in each urinary diversion case in Germany has been published.^2^ In 17 758 patients who underwent urinary diversions between 1970 and 2007, 32 cases of secondary tumors were noted; two cases occurred with ileal conduit diversion, two cases with neobladder reconstruction, 16 cases with ureterosigmoidostomy, four cases with ileocystoplasties, and the other eight cases with other types of urinary diversions. Secondary tumors did not include urothelial tumor recurrence in the upper urinary tract. The rate (0.02%) of tumor development after an ileal conduit was the lowest compared to other urinary diversions.

It is known that a GIST is the most common mesenchymal neoplasm of the gastrointestinal tract, commonly arising in stomach and small intestine.^9^ Although most GISTs do not show symptoms, large‐sized GISTs sometimes cause bleeding and abdominal pain. GISTs in the small intestine are known to be difficult to find at an early stage, but are often found after gastrointestinal bleeding.^10^, ^11^ GISTs have the potential for metastases and recurrence. The risk of metastases and recurrence is based on the diameter of the primary tumor and the mitotic count. In our case, the diameter was 5 cm and mitotic count was below 5/50 HPF, so the patient’s GIST was diagnosed as low risk.^11^, ^12^

Surgery is the standard treatment for non‐metastatic GISTs. All tumors sized more than two centimeters should be en bloc resected with their pseudocapsule in order to yield an adequate margin. Imatinib is the standard treatment for metastatic GISTs. Although GISTs responded poorly to chemotherapy or radiation therapy, tyrosine kinase inhibitors such as imatinib, were found to be effective for GISTs, being able to improve median survival.^13^

Although a GIST is a rare condition arising from urinary diversion, regular checkups may be necessary to monitor the development of this malignancy.

## Conflict of interest

The authors declare no conflict of interest.
